# Gender gap in annual preventive care services in France

**DOI:** 10.1016/j.eclinm.2022.101469

**Published:** 2022-05-27

**Authors:** Bamba Gaye, Hélène Hergault, Camille Lassale, Magalie Ladouceur, Eugenie Valentin, Maxime Vignac, Nicolas Danchin, Mor Diaw, Marina Kvaskoff, Sarah Chamieh, Frederique Thomas, Erin D. Michos, Xavier Jouven

**Affiliations:** a INSERM, U970, Paris Cardiovascular Research Center, Department of Epidemiology, Paris, France; b Université de Paris, INSERM, Paris Cardiovascular Research Centre, Paris, France; c AP-HP, Ambroise Paré Hospital, Cardiology Department, Paris, France; d Hospital del Mar Research Institute (IMIM), Barcelona, Spain; e CIBER of Pathophysiology of Obesity and Nutrition (CIBEROBN), Carlos III Health Institute, Madrid, Spain; f Preventive and Clinical Investigation Center, Paris, France; g AP-HP, Georges Pompidou European Hospital, Cardiology Department, Paris, France; h Laboratoire de Physiologie et Explorations Fonctionnelles, FMPO - UCAD, Dakar, Sénégal; i IRL3189 Environnement, santé, sociétés CNRS/UCAD Dakar/ UGB Saint-Louis/ USTTB Bamako/ CNRST Ouagadougou; j CESP, Fac. de médecine - Univ. Paris-Sud, Fac. de médecine - UVSQ, INSERM, Université Paris-Saclay, 94805, Villejuif, France^,^; k Gustave Roussy, F-94805, Villejuif, France; l Johns Hopkins Ciccarone Center for the Prevention of Cardiovascular Disease, Baltimore, Maryland, United States

**Keywords:** Preventive medicine, Cardiovascular screening, Gender gap, Women, Mortality

## Abstract

**Background:**

In France, screening for cardiovascular risk factors is recommended during annual preventive visits. However, data are lacking on the temporal trend in women's uptake to preventive care services, and in cardiovascular and mortality outcomes**.** The aim of the study was to investigate the participation and mortality of women in annual preventive care services in a major preventive medicine center in France.

**Method:**

Ee conducted repeated cross-sectional studies including a total of 366,270 individuals who had a first examination at the Centre d'Investigations Préventives et Cliniques, France, between January 1992 and December 2011.

**Findings:**

Women's participation was low below 50 years of age, then increases from 50 to 70 years, and is lower for women older than 70 years. The gap in female participation was more pronounced among individuals with high education, low social deprivation, and no depressive symptoms. Compared with the general population, the screened population had significantly lower standardized mortality ratios (SMRs) among both men and women, for all age ranges. Screened women aged 18-49 years showed a lower mortality gain compared with men of the same age; SMRs did not differ significantly by sex for individuals over 50 years.

**Interpretation:**

In this community-based sample, compared with men, women's participation to annual preventive care services was lower, and screened women had a lower mortality gain. Despite the demonstrated benefit of annual check-ups on health, there is a gender gap in adherence to preventive programs and in efficiency of screening programs, especially in the young age range. This gap in cardiovascular disease prevention may result in poorer cardiovascular health in women. Urgent adaptations to overcome this gender gap in preventive screening in France are warranted.

**Funding:**

Bamba Gaye is supported by the Fondation Recherche Médicale grant.

**Research in context**

**Evidence before the study**

A sex (biological) and gender (social/cultural) gap has been described in several domains of cardiovascular screening regarding the identification and treatment of cardiovascular disease which disproportionately favors men. Differential access to the health care system is likely to play a major role in this health gap. However, there is little data in France documenting a potential sex or gender gap in the use of preventive services, in cardiovascular risk factors and mortality.

**Added value of the study**

This study suggests that there were less women than men attending standardized medical visits in primary prevention centers. Participation was greatly dependent of age and plateaued to its lowest between 30 and 50 years old. Furthermore, even if overall mortality rates among those participating in the screening program were lower than in the general population, a lower benefit of cardiovascular screening in terms of mortality among women of reproductive age was observed.

**Implications of all the available evidence**

The reasons underlying the lower female participation in preventive care visits and especially women of reproductive age need to be further explored in future studies. Broadening the scope of action of obstetricians/gynecologists and strengthening the partnerships between obstetrics/gynecology and primary care could be a major item of an action plan. Besides, cardiovascular risk screening in its current application might not be fully adapted to women and especially young women. Urgent adaptations to overcome this gender gap in preventive screening in France are warranted.

Alt-text: Unlabelled box

## Introduction

A sex (biological) and gender (social/cultural) gap has been described in several domains of cardiovascular screening regarding the identification and treatment of cardiovascular disease (CVD) which disproportionately favors men.^1^, ^2^, ^3^ Differential access to the health care system is likely to play a major role in this health gap.^1^^,^^4^ Screening for cardiovascular risk factors is recommended through routine annual preventive visits, as early identification of risk factors and implementation of appropriate preventive treatment is recommended to mitigate the morbidity and mortality associated with CVD.^5^ In France, free standardized medical examinations subsidized by the national health insurance system for salaried workers are offered to all working and retired adult employees and their families. However, there is little data in France documenting a potential sex or gender gap in the use of preventive services, in cardiovascular risk factors and mortality.

Using data from a preventive medical center in Paris, we aimed to:^1^ describe patient characteristics and temporal trends in the participation of women in annual preventive visits;^2^ describe cardiovascular health metrics among women participating in these annual preventive screening visits; and^3^ assess sex-specific mortality rates.

## Methods

### Study design

The present study population consisted of individuals who were examined at the *Centre d'Investigations Préventives et Cliniques* (IPC Centre), a preventive medical center in Paris, one of the largest preventive medical centers in France that conducts 20,000-25,000 examinations/year.^6^ The catchment area of the IPC Centre is the city of Paris and its surrounding suburbs, an area covering 11 million inhabitants. All participants belonging to the French general healthcare scheme (*régime général*) for employees, covering over 80% of the adult population, were available for this study.^7^ Of those covered by the healthcare system, 48.9% are males and 51.5% were females, a representative proportion to the total population. There were 369,277 individuals who had an examination at the IPC Centre between January 1992 and December 2011, and we excluded 3007 patients because they had history of coronary heart disease or stroke, leading to a study population of 366,270 individuals. Medical, psychological, and socioeconomic data were collected during the examination.

For the purposes of this study, we considered sex/gender as binary (women vs men) obtained by self-identification; data for transgender individuals were not available.

All participants provided written informed consent for their data to be used for the epidemiological study^8^ and the IPC Centre received authorization for epidemiological data analyses from the French National Commission on Data Protection and Privacy (*Commission Nationale de l'Informatique et des Libertés*). Details on data collection have been previously described.^8^

### Covariates

Self-administered questionnaires were used to assess socio-economic status, depressive symptoms, medical history and medication use. Socio-economic status was estimated by two measures: education level (available since 1997 only; divided into low [no or primary education], intermediate [secondary education], and high [higher education or university]) and the EPICES deprivation score^9^ (available since 2002). The EPICES deprivation score is a summary measure of socio-economic deprivation and has been validated in France in a sample of 200,000 persons against two other indices of deprivation, i.e. the Townsend and the Carstairs indexes.^9^ The EPICES deprivation score includes 11 items on marital status, health insurance coverage, socio-economic status, family support, and leisure and recreational activities (the full questionnaire is provided in **e-Table 1**). A positive response to an item was attributed a weight corresponding to the regression coefficient, whereas a negative response was attributed a weight of 0.^9^ The score was obtained by adding each weight to the intercept and varied from 0 to 100, with higher scores indicating lower socio-economic status (high social deprivation).^9^ Subjects with a score over 30 were considered socio-economically deprived.^9^ Depressive symptoms were assessed using the 13-item Questionnaire of Depression 2^nd^ version, Abridged (QD2A).^10^ Participants with a score ≥7 on the QD2A or who were on antidepressants were referred to as having depressive symptoms.^10^

### Measurement and definitions of cardiovascular profiles

We used 6 of the Life's simple 7 metrics proposed by the American Heart Association (AHA) to measure cardiovascular health^11^ Body mass index (BMI) was calculated from weight and height measurements obtained using calibrated scales and a wall mounted stadiometer, respectively. An ideal body weight was defined as a BMI <25 kg/m^2^. Smoking habits were assessed by a standardized questionnaire, and ideal smoking was defined as not smoking (never smoked or quit smoking >12 months). Physical activity was measured by a standardized questionnaire about time spent walking per day. Ideal physical activity was defined as walking ≥1 hour/day. Blood pressure (taken on the sphygmomanometer calibrated with digital readings) was measured with a manual mercury sphygmomanometer between January 1992 and July 1998, and with a validated digital blood pressure device (TM-2541, A&D Company, Tokyo, Japan) between July 1998 and December 2011. At each examination, blood pressure was measured three times on the right arm in the supine position after a 10-minute rest. The mean of the last two measurements was used in the analyses. Ideal blood pressure was defined as untreated Systolic/Diastolic blood pressure of <120/80 mmHg. Lipid profile and glucose were measured following an overnight fast. Ideal total cholesterol was defined as untreated values of <200 mg/dl (to convert cholesterol to mmol/L, multiply values by 0.0259); and ideal fasting plasma glucose as untreated values of <100 mg/dl (to convert glucose to mmol/L, multiply by 0.0555). When risk factors were diagnosed, counseling and treatments according to actual recommendations were given.

### Ascertainment of mortality

Vital status was obtained for all individuals from the French National Registry of Deaths (*Institut National de Statistiques et d'Etudes Economiques* (INSEE), Paris) between January 1^st^, 1992 and December 31^st^, 2016.

### Statistics

The study flowchart is described in **e-Fig. 1**. We described patient characteristics and prevalence of ideal CVH metrics for 4 pre-specified examination periods spanning 5 years: 1992-1996, 1997-2001, 2002-2006, and 2007-2011. New participants were included at each examination session.

Our primary outcome was the proportion of women attending annual health visits. We used logistic regression analyses to test for differences in the percentage of women across the examination periods by education level, EPICES deprivation score, and depressive symptoms. In addition, we also examined the age trends in the proportion of women attending annual health visits. We used demographic data from the INSEE to normalize the sex ratio of our population to the French age and sex structure of 1992 to 2011.

Our secondary outcome was the prevalence of ideal CVH metrics. We used logistic regression to assess the difference in proportions meeting ideal CVH measures between men and women. Individuals were matched by age and depression status (Population 2), and the models were adjusted for age, depression, EPICES deprivation score, and the 6 CVH metrics over the 5-year examination periods.

Our third outcome was subsequent mortality among patients participating in annual preventive care exams. For this, we first computed standardized mortality ratios (SMRs) by age groups. An SMR is the ratio of an observed (n) to an expected number of deaths (A). The expected number of deaths is obtained on the basis of a reference population death structure (standard mortality). An SMR greater (or lesser) than 1 indicates higher (or lower) mortality in the study area compared with the reference population. The 95% confidence intervals of the SMRs were calculated using the Byar method as presented in Breslow-Day.^12^ Observed number of deaths was the observed number of deaths in our study population. Expected number of deaths was the number of deaths in the French population of same age and sex (data originated from INSEE).

We computed the multivariable-adjusted hazard ratio of the association between sex and all-cause mortality using Cox proportional hazards regression models adjusted for age, depression, EPICES deprivation score, and the 6 CVH metrics over the 5-year examination periods.

Two-tailed P values of less than 0.05 were considered statistically significant. Statistical analyses were undertaken using SAS software version 9.4 (SAS Institute Inc., Cary, NC) and R software, version 3.3.2.

### Role of the funding source

The funding body had no role in the study design, collection, analysis, and interpretation of data; in the writing of the manuscript; and in the decision to submit the manuscript for publication. All the authors accessed the data and decided to submit the manuscript for publication.

## Results

**Figure 1:**
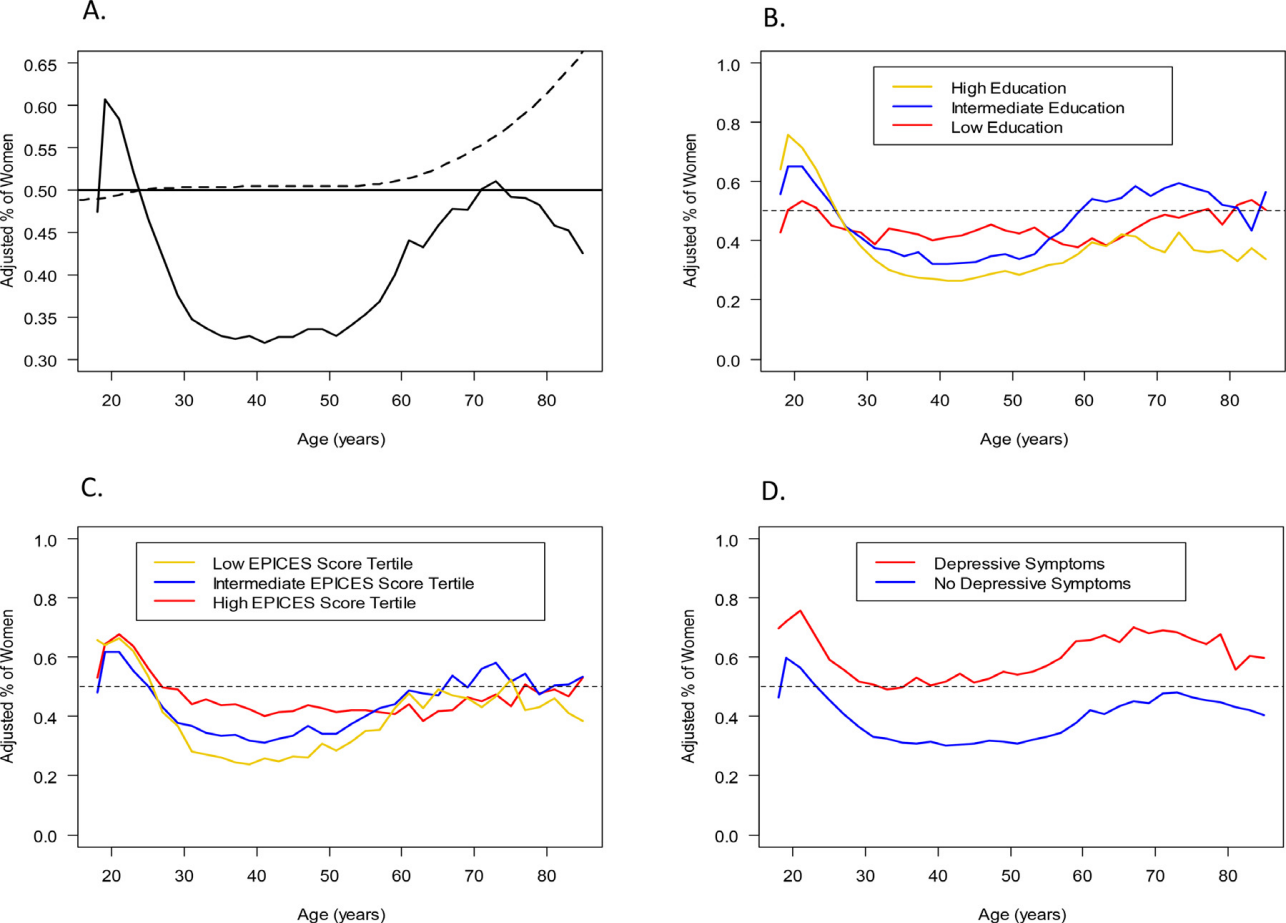
**Age trends in the proportion of women in the study by age, adjusted for the age and sex structure of the French population.** A: Overall; B: According to education level; C: According to EPICES deprivation score; D: According to depressive symptoms. The dashed line in (A) should correspond to “unadjusted percentage”.

**Figure 2:**
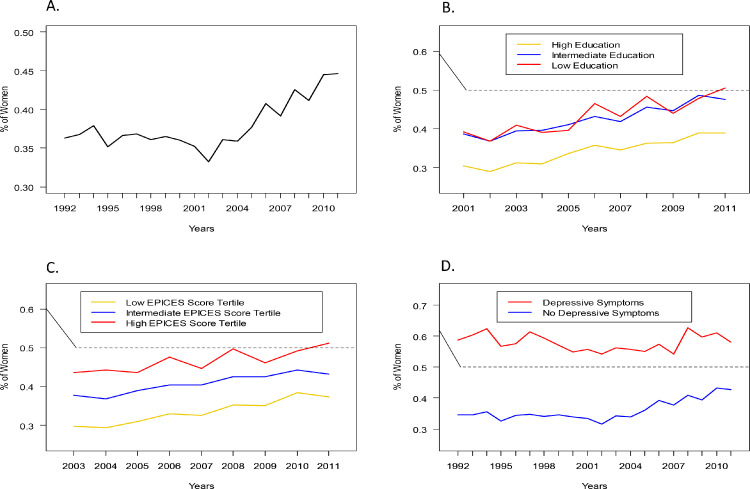
**Temporal trends in the proportion of women in the study per year.** A: Overall; B: According to education level; C: According to EPICES deprivation score; D: According to depressive symptoms. IPC: Centre d'Investigations Préventives et Cliniques.

Among the 366,270 included participants who attended annual preventive care visits during the study period of 1992-2011, 37.7% (138,228) were women. The mean age was 44.7 (SD 13) years (44.9 ± 14.4 in women vs. 44.6 ± 12.2 in men, p <0.001). Between the ages of 18 and 24 years old, a higher percentage of women were examined at the IPC center compared with men (Figure 1A). However, among participants between 24 and 60 years old, the proportion of examined women represented only 33.9% (89,964). There was a decline in the rate of women's participation from 24 years onwards, which plateaued to its lowest rate between 30 and 50 years of age. Thereafter, the proportion of women attending the visit increased and reached 50% around the age of 70 years, after which it declined again until beyond the age of 80. Between 1992 and 2011, the percentage of women evaluated for preventive care increased overall, but it remained lower than 50% at all time points (Figure 2A).

In subgroup analyses, the previously described age and sex trends in the proportion of individuals attending annual preventive visits were differently and linearly associated with education level and EPICES deprivation score: the gap in female participation was more pronounced among individuals with high education (Figure 1B) or low social deprivation (Figure 1C). However, female participation was higher in those with depressive symptoms (Figure 1D). While we observed similar findings across study years for education (Figure 2B) and social deprivation (Figure 2C), global patterns in women's participation by age and across study years did not differ according to depressive status (Figure 2D).

We then examined the proportion of individuals meeting ideal CVH metrics by sex (Table 1) and by age (**e-Table 2**). We found that compared with men, women had lower physical activity levels but were less likely to be current or former smokers, and more likely to have ideal BMI, total cholesterol, fasting glucose, and blood pressure levels. Of note, however, the proportion of obese women was slightly higher than that of obese men (11.9% and 9.7% respectively). Moreover, the proportion of individuals with an ideal BMI dropped more strongly after 50 years old in women compared to men. Women were also more likely to have depressive symptoms and a high deprivation score, but less likely to have a high education level compared with men. Temporal trends of cardiovascular risk factors are described in **e-Table 3**. Whilst the proportion of men with an ideal BMI remained roughly stable across study periods, the proportion of women dropped from 72% in 1992-96 to 56% in 2007-2011.

**Table 1:** Cardiovascular health metrics by sex^a^.

	Women	Men	P-value for interaction
N (%)	103,668 (38%)	168,884 (62%)	
**Smoking**			<.0001
Poor: Current	24,811 (23.97%)	55,471 (32.90%)	(ref)
Intermediate: Former or quit ≤12m	1923 (1.86%)	4751 (2.82%)	<.0001
Ideal: Never or quit >12m ago	76,760 (74.17%)	108,362 (64.28%)	0.0004
**Body Mass Index**			<.0001
Poor: ≥30 kg/m^2^	12,316 (11.97%)	16,289 (9.70%)	(ref)
Intermediate: 25-29.9 kg/m^2^	23,597 (22.93%)	66,275 (39.45%)	<.0001
Ideal: <25 kg/m^2^	66,990 (65.10%)	85,427 (50.85%)	0.0051
**Physical Activity**			
Poor: No or walking <1 h/day	62,937 (60.72%)	92,609 (54.85%)	(ref)
Ideal: Walking ≥1 h/day	40,709 (39.28%)	76,245 (45.15%)	<.0001
**Total cholesterol**			<.0001
Poor: >6.138 mmol/L	24,236 (23.54%)	43,794 (26.09%)	(ref)
Intermediate: 5.136 – 6.138 mmol/L	37,421 (36.34%)	61,752 (36.79%)	<.0001
Ideal: <5.136 mmol/L	41,321 (40.13%)	62,320 (37.12%)	<.0001
**Fasting glucose**			<.0001
Poor: >6.938 mmol/L	2,041 (1.98%)	5,896 (3.51%)	(ref)
Intermediate: 5.55- 6.938 mmol/L	25,081 (24.33%)	68,143 (40.57%)	0.02
Ideal: <5.55 mmol/L	75,946 (73.69%)	93,928 (55.92%)	<.0001
**Blood Pressure**			<.0001
Poor: ≥140/90 mmHg	21,545 (21.46%)	47,770 (29.15%)	(ref)
Intermediate: 120-139/80-89 mmHg	42,343 (42.18%)	85,336 (52.07%)	<.0001
Ideal: <120/80 mmHg	36,508 (36.36%)	30,785 (18.78%)	<.0001
**Depressive symptoms: Yes**	11,820 (11.40%)	9052 (5.36%)	<.0001
Education level*			<.0001
Low	11,165 (19.84%)	14,537 (15.77%)	(ref)
Intermediate	24,306 (43.20%)	35,023 (38.00%)	<.0001
High	20,793 (36.96%)	42,616 (46.23%)	<.0001
EPICES deprivation score*			<.0001
Low	14,298 (32.90%)	29,606 (41.80%)	(ref)
Intermediate	12,394 (28.52%)	19,948 (28.30%)	<.0001
High	16,762 (38.57%)	21,281 (30.04%)	<.0001

SI conversion factors: To convert total cholesterol values to mmol/L, multiply by 0.0259; to convert glucose values to mmol/L, multiply by 0.0555

a Individuals were matched by age and depression status.

⁎ Education and EPICES deprivation score were available from 2001 and 2003, respectively. Note: All individuals with available cardiovascular health metrics were included in trend analyses for each specific metric; therefore, sample sizes might vary by cardiovascular health metrics. CVH = cardiovascular health.

Furthermore, we observed the percentage of women with and without children by age groups attending IPC visits (**e-Table 4**). Among women aged 30-39 years (average pregnancy age in France) who attended IPC visits, 60.1% reported having children. This percentage increases to 81.1% and 85.0% in the 40 to 49 and 50 to 59 age range.

Finally, we examined associations of SMRs by sex (Table 2). Among the participants undergoing screening, we observed a significantly lower mortality compared with the general population (SMR<1) in both genders across all age groups at inclusion. However, between 18 and 49 years of age, the magnitude of mortality gain was lower among women than in men, (SMR 0.83 [95% confidence interval: 0.75-0.92] in women vs. 0.70 [0.66-0.74] in men). SMRs did not substantially differ by sex for individuals included after the age of 49 years old. Kaplan-Meier curves estimating all-cause mortality on average across ages and over all the study period show than women had lower incidence of mortality than men (**e-Figure 3), which was confirmed in Cox models** adjusted for covariates: 0.82 [0.75 - 0.90] (**e-Table 5).**

**Table 2:** Mortality rates per 1000 person-years^a^.

	Whole Population			Women			Men		
Age* range (years)	French Population	IPC Population	Standardized mortality ratio	French Population	IPC Population	Standardized mortality ratio	French Population	IPC Population	Standardized mortality ratio
[18-49]	0.94	0.76	0.81 [0.76 – 0.85]	0.61	0.51	0.83 [0.75 – 0.92]	1.28	0.90	0.70 [0.66 – 0.74]
[50-54]	3.34	1.94	0.58 [0.55 – 0.62]	2.25	1.21	0.54 [0.47 – 0.61]	4.48	2.33	0.52 [0.49 – 0.56]
[55-59]	5.40	3.06	0.57 [0.54 – 0.60]	3.50	1.89	0.54 [0.49 – 0.60]	7.41	3.73	0.50 [0.48 – 0.53]
[60-64]	7.90	4.22	0.53 [0.51 – 0.56]	4.94	2.52	0.51 [0.46 – 0.56]	11.13	5.31	0.48 [0.45 – 0.50]
[65-69]	11.03	5.32	0.48 [0.46 – 0.50]	7.05	3.30	0.47 [0.43 – 0.51]	15.44	6.73	0.44 [0.41 – 0.46]
[70-74]	16.16	8.38	0.52 [0.50 – 0.54]	10.85	5.52	0.51 [0.47 – 0.55]	22.30	10.62	0.48 [0.45 – 0.50]
[75-79]	23.91	8.38	0.35 [0.33 – 0.37]	17.12	5.92	0.35 [0.32 – 0.38]	32.69	10.47	0.32 [0.30 – 0.34]
>79	90.16	36.98	0.41 [0.40 – 0.42]	82.79	30.78	0.37 [0.36 – 0.39]	103.77	44.51	0.43 [0.41 – 0.45]

⁎ Age at inclusion in the cohort.

a Individuals were matched by age and depression status.

## Discussion

We found that overall there were less women than men attending standardized medical visits in France primary prevention centers (37.8%). Although the participation of women remains low overall, we observed an increase over the study period, which is probably due to public health campaigns and initiatives for CVD risk awareness that began in the 1990s.^2^

Contrasting our results, the French data from Baromètre Santé reported women participation in CVD preventive care services was equivalent to that of men (51%) in 2000.^13^ This may be explained by the different mode of recruitment (telephone survey in the Barometre Santé) and the nationwide compared to Paris only in our study.^13^

In international studies, precise data regarding female participation in preventive care depending on age and year are scarce.^14^, ^15^, ^16^ In INTERHEART, participation of women varied between countries. In Western Europe, a slightly lower participation of women was observed.^14^ Other studies suggest a lower participation among men in preventive medicine^17^ in general. The type of screening (e.g., general health check, colorectal cancer, cardiovascular risk factors) seems to have impact on participation among men and women and could explain the discordance with our results.^18^

As previously described, differences in behaviors between men and women regarding cardiovascular care are a major mechanism of gender gap in CVD.^19^ While some of the behavioral barriers affecting women's participation in secondary care, such as cardiac rehabilitation, have already been identified, encompassing health beliefs, religious reasons, lack of family, financial, or logistical support, and underestimation of risk,^19^^,^^20^ the reasons explaining women's lower participation to preventive programs remains to be further investigated.

Regarding sex differences in cardiovascular risk factors, our results are concordant with other French studies where women smoke less and have a better blood pressure control than men.^21^ The higher proportion of obese individuals among women is also observed in the French CARVAR study population.^21^ Biological sex and gender related behavior appear to be partially explaining those findings.

Although we do not directly have information on this in our study, a potential mechanism underlying this gender gap may be women's access to screening through regular obstetric/gynecologist visits during their childbearing years. In France, women have access to free cervical cancer screening between 25 and 65 years of age, with a high participation rate of 61%.^22^ Although this is not a proven trend, we hypothesize that the contrast between accessibility to other preventive care such obstetrician/gynecological services and accessibility to global health checks for cardiovascular risk screening, despite the former knowingly insufficient to replace the latter, might explain why women may be less likely than men to seek a medical follow-up to assess their health.

Moreover, although data suggests that heart disease is the leading cause of death for women, it is largely underrecognized by both men and women.^23^ Cardiovascular risk is underestimated while the risk of cancer, especially breast or cervical cancer, continues to dominate health interventions and disease prevention.^24^ This wrongly perceived risk might make women favor completing their gynecological follow-up while declining a cardiovascular preventive visit.^24^ Along the same lines, the increase in female participation after age 50 observed in our study coincides with the decline of cervical cancer screening rates after age 50.^25^ In addition, the age of 50 corresponds to the mean menopausal age, which is associated with a decrease in the rate of gynecologist visits.

Another hypothesis could be linked to motherhood. Indeed, child caring might refrain mothers to seek healthcare, especially for women who work.^26^ Bernstein demonstrated that motherhood, when associated with stressors (low financial resources, being a single mother, full time employment, etc.), was associated with worse health outcomes.^27^ Nevertheless, some data are discordant with those results. The role accumulation theory suggests that taking on multiple responsibilities (such as in motherhood) may actually positively enhance one's sense of self-worth^28^ and encourage to take screening.

Another finding is that the proportion of women taking up the CVD screening was lower in the high education and low social deprivation groups. A potential explanation of this finding could be that women with a higher socioeconomic status have higher rates of participation in cervical cancer screening and are also likely to have more frequent visits to gynecologist and other specialist doctors.^25^

In our study, participation in cardiovascular screening was associated with benefits for both men and women. Mortality rates among those participating in the screening program were lower than in the general population, regardless of the age at screening. This result is concordant with numerous reports on the beneficial effect of cardiovascular prevention and screening on mortality.^29^ The mortality difference with the general population, however, increased proportionately with age at screening in both male and female populations. Thus, CVD screening may prevent more events in an older than in a younger population. This is also related to the length of follow-up. Prevention actions on cardiovascular risk factors among young people have a delayed impact. The length of follow-up was likely insufficient in this young population to capture a difference in mortality. A longitudinal study could further develop these findings by identifying the long-term impact of preventive care among the younger participants.

Additionally, we also noted that the mortality benefit associated with screening (compared with the general population) was lower among young women (18-49 years old) compared with young men. Three hypotheses may be developed. First, cardiovascular mortality accounts for less deaths in young women than in young men.^30^ Consequently, women may appear to have a lower benefit from CVD screening in the short-term but benefit over long-term follow-up. Second, growing evidence demonstrates that young women have sex-specific nontraditional atherosclerotic risk factors such as preterm delivery,^31^ hypertensive disorders of pregnancy,^32^ gestational diabetes^33^ or auto-immune disease.^34^ These risk factors might not be well targeted by classic cardiovascular screening.^35^ Finally, screening of traditional cardiovascular risk factors might be performed with less consistency and recommendations are less likely adopted among young women. Indeed, in the female population, especially young females, prescriptions of appropriate preventive medicine is less frequent^36^ and less followed.^37^^,^^38^

Primary care exams are important for CVH and other health screening. Depending on the setting, the following metrics may be assessed: vital signs (temperature, blood pressure, heart rate, body mass index), overall appearance, review of symptoms, family history, smoking and other substance use history, heart exam, lung exam, neurological exam, dermatological exam, head and neck exam, extremities exam, and breast exam (for women), if not performed by a gynecologist. Additionally, traditional cardiovascular risk factors, such as blood lipids, should be ascertained, and assessment of cardiovascular risk using the SCORE risk calculator should be used for those over age 40.^39^ The need of a yearly gynecologic exam is justified by the need of specialists (gynecologists) with wider expertise in fertility issues, birth control, cancer prevention and sexually-transmitted infections. In fact, studies have demonstrated that obstetricians/gynecologists are a major gateway into women's care and can positively influence a woman's lifetime health.^40^ In France, many gynecologists have become accustomed to screening for other health factors such as cardiovascular risk and referring to primary care as appropriate. This approach may help to overcome the potential hazard associated with the low participation of women in annual preventive care service. This may, however, require assurance that their workload does not lead to providing low levels of screening or health counseling.

Additionally, our results suggest a lower benefit of cardiovascular screening in terms of mortality among women of reproductive age. Therefore, usual cardiovascular programs should be tailored to take into consideration the specific somatic and psychological risk factors for women. This might improve cardiovascular screening efficiency.

We used a large database of over 360,000 participants in a CVD preventive screening program to examine trends in participation over time from 1992 to 2011 by sex and in relation to age, sociodemographic and depressive factors. Moreover, we were able to compare mortality rates of participants in this screening program compared with the general population, and to examine differences by sex. However, our study has some notable limitations: it relates to a single center in France and do not reflect other preventive screening programs in other parts of the country, or the world. Race/ethnicity was omitted from the study due to the lack of data availability in France. We also did not have information on time spent on domestic activities, which could inform on women's ability to actually access services, or on history of seeking screening elsewhere. Among other unmeasured confounding factors, these important factors may explain some of the gender gap observed and would have helped our understanding of women's care seeking over the life course. Moreover, gender was considered only binary and we cannot present any results on the transgender and non-binary population. In addition, the reasons for non-participation was not possible to determine and can only be speculated. Due to the voluntary basis of the IPC visits, those who did participate were more likely to want to work towards an ideal CVH, adhere to treatment and improve their overall health independently from the preventive care services, creating a sample selection bias. Furthermore, self-administered questionnaires were used to assess socio-economic status, depressive symptoms, medical history, and medication use in this study. This leads to possible reporting bias and may result in missing data elements and incomplete data for reported cases. Finally, factors such as seeking other forms of care aside from preventive care could not be taken into account in this study and may have influenced the mortality estimates and thus, interpretation of the SMR has to be done with caution.

In conclusion, despite the demonstrated benefit of annual check-ups on health, there was a gender gap in adherence and efficiency of preventive programs in our study, which disfavors women. One of the potential mechanisms underlying this gender gap may include women's access to screening through regular gynecologist visits, which are known to be insufficient to replace global health checks. The reasons underlying the lower female participation in preventive care visits need to be further explored. Broadening the scope of action of obstetricians/gynecologists and strengthening the partnerships between obstetrics/gynecology and primary care could be a major item of this action plan. Besides, cardiovascular risk screening in its current application might not be fully adapted to women and especially young women. Urgent adaptations to overcome this gender gap in preventive screening in France are warranted.

## Declaration of interests

The authors have nothing to disclose.

## Appendix Supplementary materials

Image, application 1
