# Supplementary material for: Gender gap in annual preventive care services in France
Source: eClinicalMedicine. 2022 May 27;49:101469. doi: 10.1016/j.eclinm.2022.101469 (PMC9156877; doi:10.1016/j.eclinm.2022.101469)
Supplement: Supplementary file 1 [file mmc1.docx]

**Supplemental Data**

**e-Table 1.** EPICES score (*Evaluation de la Précarité et des Inégalités de santé dans les Centres d’Examens de Santé* - Evaluation of Deprivation and Inequalities in Health Examination Centres)

**e-Table 2.** Prevalence of cardiovascular disease risk factors by age and sex in the study population (n=272,521)

**e-Table 3.** Temporal trends in the prevalence of factors associated with cardiovascular health by sex and examination period

**e-Table 4.**  Percentage of women with and without children by age groups attending IPC visits

**e-Table 5.** HRs of women for All-Cause Mortality: analysis by age group

**e-Figure 1.** Flowchart of sample selection

**e-Figure 2.** Flowchart of sample selection matched by age and depressive status

**e-Figure 3.** Kaplan Meier curves denoting association of sex with all-cause mortality

**e-Table 1.** EPICES score (*Evaluation de la Précarité et des Inégalités de santé dans les Centres d’Examens de Santé* - Evaluation of Deprivation and Inequalities in Health Examination Centres)

| **Questions** | **Score** |  |
| --- | --- | --- |
|  | **Yes** | **No** |
| 1. Do you sometimes meet with a social worker (welfare worker, educator)? | 10.06 | 0 |
| 2. Do you have complementary health insurance (mutual insurance)? | -11.83 | 0 |
| 3. Do you live as a couple? | -8.28 | 0 |
| 4. Are you a homeowner or will you be one in the near future? | -8.28 | 0 |
| 5. Are there periods in the month when you have real financial difficulties in facing your needs (food, rent, electricity)? | 14.80 | 0 |
| 6. Have you participated in any sports activities in the last 12 months? | -6.51 | 0 |
| 7. Have you gone to any shows (cinema, theatre) in the last 12 months? | -7.10 | 0 |
| 8. Have you gone on holiday during the past 12 months? | -7.10 | 0 |
| 9. Have you seen any family members in the past six months (other than your parents or children)? | -9.47 | 0 |
| 10. If you were in need or trouble (financial, familial, or health-related), is there anyone around you who could host you for a few days? | -9.47 | 0 |
| 11. If you were in need or trouble (financial, familial, or health-related), is there anyone around you who could help you financially (material aid such as lending you money)? | -7.10 | 0 |
| Intercept | 75.14 | 0 |

**e-Table 2.** Prevalence of cardiovascular disease risk factors by age and sex in the study population (n=272,521)^a^

|  | **Women: 103,668 (38%)** | | | | | | | | **Men: 168,884 (62%)** | | | | | | | | **P-value Interaction for sex** |
| --- | --- | --- | --- | --- | --- | --- | --- | --- | --- | --- | --- | --- | --- | --- | --- | --- | --- |
| **Age (years)** | **[18 - 49]** | **[50 - 54]** | **[55 - 59]** | **[60 - 64]** | **[65 - 69]** | **[70 - 74]** | **[75 - 79]** | **≥80** | **[18 - 49]** | **[50 - 54]** | **[55 - 59]** | **[60 - 64]** | **[65 - 69]** | **[70 - 74]** | **[75 - 79]** | **≥80** |  |
| **N (%)** | 62199 (60.00%) | 13150 (12.68%) | 11298 (10.90%) | 8393 (8.10%) | 3939 (3.80%) | 2579 (2.49%) | 1524 (1.47%) | 586 (0.57%) | 112585 (66.66%) | 21707 (12.85%) | 15556 (9.21%) | 11013 (6.52%) | 4063 (2.41%) | 2336 (1.38%) | 1170 (0.69%) | 454 (0.27%) |  |
| **Sex (%)** | 62199 (35.59%) | 13150 (37.73%) | 11298 (42.07%) | 8393 (43.25%) | 3939 (49.23%) | 2579 (52.47%) | 1524 (56.57%) | 586 (56.35%) | 112585 (64.41%) | 21707 (62.27%) | 15556 (57.93%) | 11013 (56.75%) | 4063 (50.77%) | 2336 (47.53%) | 1170 (43.43%) | 454 (43.65%) | <.0001 |
| **Smoking** |  |  |  |  |  |  |  |  |  |  |  |  |  |  |  |  | <.0001 |
| Poor: | 18900 (30.44%) | 2512 (19.14%) | 1765 (15.65%) | 954 (11.39%) | 364 (9.25%) | 183 (7.10%) | 100 (6.57%) | 33 (5.64%) | 41632 (37.05%) | 6398 (29.52%) | 4029 (25.95%) | 2247 (20.44%) | 658 (16.21%) | 336 (14.40%) | 124 (10.63%) | 47 (10.38%) | (ref) |
| Intermediate: | 1432 (2.31%) | 166 (1.27%) | 145 (1.29%) | 102 (1.22%) | 32 (0.81%) | 30 (1.16%) | 12 (0.79%) | 4 (0.68%) | 3485 (3.10%) | 518 (2.39%) | 367 (2.36%) | 229 (2.08%) | 88 (2.17%) | 35 (1.50%) | 21 (1.80%) | 8 (1.77%) | 0.44 |
| Ideal: | 41764 (67.26%) | 10444 (79.59%) | 9368 (83.06%) | 7323 (87.40%) | 3539 (89.94%) | 2363 (91.73%) | 1411 (92.65%) | 548 (93.68%) | 67260 (59.85%) | 14760 (68.09%) | 11129 (71.68%) | 8517 (77.48%) | 3314 (81.63%) | 1963 (84.10%) | 1021 (87.56%) | 398 (87.86%) | <.0001 |
| **Body Mass Index** |  |  |  |  |  |  |  |  |  |  |  |  |  |  |  |  | <.0001 |
| Poor: | 6130 (9.95%) | 1995 (15.24%) | 1741 (15.48%) | 1158 (13.86%) | 608 (15.51%) | 409 (15.90%) | 208 (13.73%) | 67 (11.55%) | 8832 (7.89%) | 2616 (12.08%) | 2148 (13.86%) | 1560 (14.22%) | 581 (14.36%) | 342 (14.74%) | 153 (13.17%) | 57 (12.67%) | (ref) |
| Intermediate: | 11447 (18.58%) | 3471 (26.52%) | 3190 (28.36%) | 2597 (31.08%) | 1316 (33.58%) | 878 (34.12%) | 512 (33.80%) | 186 (32.07%) | 39285 (35.11%) | 10048 (46.41%) | 7532 (48.60%) | 5410 (49.33%) | 2061 (50.95%) | 1135 (48.90%) | 589 (50.69%) | 215 (47.78%) | <.0001 |
| Ideal: | 44045 (71.48%) | 7624 (58.24%) | 6318 (56.16%) | 4600 (55.06%) | 1995 (50.91%) | 1286 (49.98%) | 795 (52.48%) | 327 (56.38%) | 63779 (57.00%) | 8987 (41.51%) | 5819 (37.54%) | 3997 (36.45%) | 1403 (34.68%) | 844 (36.36%) | 420 (36.14%) | 178 (39.56%) | <.0001 |
| **Physical Activity** |  |  |  |  |  |  |  |  |  |  |  |  |  |  |  |  | <.0001 |
| Poor: | 38333 (61.64%) | 8268 (62.90%) | 6879 (60.89%) | 4671 (55.67%) | 2147 (54.53%) | 1416 (54.91%) | 846 (55.51%) | 377 (64.33%) | 61308 (54.47%) | 12560 (57.87%) | 8970 (57.67%) | 5822 (52.87%) | 2005 (49.36%) | 1115 (47.75%) | 599 (51.20%) | 230 (50.66%) | (ref) |
| Ideal: | 23853 (38.36%) | 4877 (37.10%) | 4419 (39.11%) | 3720 (44.33%) | 1790 (45.47%) | 1163 (45.09%) | 678 (44.49%) | 209 (35.67%) | 51256 (45.53%) | 9143 (42.13%) | 6584 (42.33%) | 5190 (47.13%) | 2057 (50.64%) | 1220 (52.25%) | 571 (48.80%) | 224 (49.34%) | <.0001 |
| **Total cholesterol** |  |  |  |  |  |  |  |  |  |  |  |  |  |  |  |  | <.0001 |
| Poor: | 7522 (12.16%) | 4195 (32.14%) | 4633 (41.30%) | 3763 (45.14%) | 1900 (48.64%) | 1212 (47.66%) | 727 (48.15%) | 284 (49.13%) | 24258 (21.65%) | 7707 (35.80%) | 5623 (36.46%) | 3779 (34.62%) | 1276 (31.67%) | 718 (31.11%) | 319 (27.55%) | 114 (25.39%) | (ref) |
| Intermediate: | 20078 (32.47%) | 5572 (42.69%) | 4746 (42.30%) | 3483 (41.78%) | 1594 (40.81%) | 1074 (42.23%) | 619 (40.99%) | 255 (44.12%) | 38235 (34.12%) | 8647 (40.16%) | 6376 (41.34%) | 4849 (44.42%) | 1833 (45.50%) | 1045 (45.28%) | 559 (48.27%) | 208 (46.33%) | <.0001 |
| Ideal: | 34235 (55.37%) | 3284 (25.16%) | 1840 (16.40%) | 1090 (13.08%) | 412 (10.55%) | 257 (10.11%) | 164 (10.86%) | 39 (6.75%) | 49560 (44.23%) | 5175 (24.04%) | 3424 (22.20%) | 2289 (20.97%) | 920 (22.83%) | 545 (23.61%) | 280 (24.18%) | 127 (28.29%) | <.0001 |
| **Fasting glucose** |  |  |  |  |  |  |  |  |  |  |  |  |  |  |  |  | <.0001 |
| Poor: | 533 (0.86%) | 364 (2.79%) | 371 (3.30%) | 317 (3.80%) | 189 (4.83%) | 143 (5.60%) | 79 (5.21%) | 45 (7.77%) | 1912 (1.71%) | 1135 (5.27%) | 1110 (7.19%) | 909 (8.31%) | 435 (10.78%) | 232 (10.03%) | 124 (10.70%) | 39 (8.65%) | (ref) |
| Intermediate: | 10170 (16.44%) | 4091 (31.32%) | 4021 (35.80%) | 3119 (37.36%) | 1644 (42.01%) | 1106 (43.30%) | 652 (43.04%) | 278 (48.01%) | 39580 (35.31%) | 10793 (50.10%) | 8013 (51.89%) | 5702 (52.14%) | 2096 (51.93%) | 1163 (50.28%) | 564 (48.66%) | 232 (51.44%) | <.0001 |
| Ideal: | 51160 (82.70%) | 8607 (65.89%) | 6841 (60.90%) | 4913 (58.85%) | 2080 (53.16%) | 1305 (51.10%) | 784 (51.75%) | 256 (44.21%) | 70596 (62.98%) | 9615 (44.63%) | 6318 (40.92%) | 4325 (39.55%) | 1505 (37.29%) | 918 (39.69%) | 471 (40.64%) | 180 (39.91%) | <.0001 |
| **Blood Pressure** |  |  |  |  |  |  |  |  |  |  |  |  |  |  |  |  | <.0001 |
| Poor: | 6264 (10.41%) | 3354 (26.29%) | 3713 (33.84%) | 3312 (40.71%) | 1950 (50.89%) | 1520 (60.90%) | 997 (67.23%) | 435 (78.38%) | 22798 (20.89%) | 7852 (37.17%) | 6772 (44.80%) | 5514 (51.42%) | 2273 (57.66%) | 1442 (63.22%) | 797 (69.97%) | 322 (72.85%) | (ref) |
| Intermediate: | 24717 (41.08%) | 6085 (47.70%) | 5067 (46.19%) | 3582 (44.03%) | 1517 (39.59%) | 839 (33.61%) | 426 (28.73%) | 110 (19.82%) | 61280 (56.16%) | 10366 (49.07%) | 6784 (44.88%) | 4334 (40.42%) | 1448 (36.73%) | 722 (31.65%) | 292 (25.64%) | 110 (24.89%) | 0.48 |
| Ideal: | 29186 (48.51%) | 3318 (26.01%) | 2191 (19.97%) | 1241 (15.26%) | 365 (9.53%) | 137 (5.49%) | 60 (4.05%) | 10 (1.80%) | 25044 (22.95%) | 2907 (13.76%) | 1561 (10.33%) | 875 (8.16%) | 221 (5.61%) | 117 (5.13%) | 50 (4.39%) | 10 (2.26%) | <.0001 |
| **Depressive symptoms: Yes** | 6815 (10.96%) | 1561 (11.87%) | 1516 (13.42%) | 928 (11.06%) | 432 (10.97%) | 258 (10.00%) | 204 (13.39%) | 106 (18.09%) | 6005 (5.33%) | 1248 (5.75%) | 942 (6.06%) | 452 (4.10%) | 198 (4.87%) | 106 (4.54%) | 72 (6.15%) | 29 (6.39%) | 0.09 |
| **Education level*** |  |  |  |  |  |  |  |  |  |  |  |  |  |  |  |  | <.0001 |
| Low | 5784 (17.15%) | 1717 (24.25%) | 1429 (22.77%) | 890 (19.44%) | 590 (28.45%) | 393 (28.46%) | 246 (29.32%) | 116 (37.66%) | 7753 (12.62%) | 1988 (16.81%) | 1896 (22.00%) | 1518 (25.25%) | 738 (34.36%) | 382 (30.39%) | 193 (29.92%) | 69 (28.99%) | (ref) |
| Intermediate | 13801 (40.92%) | 3049 (43.07%) | 2986 (47.59%) | 2282 (49.84%) | 925 (44.60%) | 703 (50.91%) | 419 (49.94%) | 141 (45.78%) | 23119 (37.63%) | 4960 (41.95%) | 3402 (39.47%) | 2118 (35.24%) | 653 (30.40%) | 457 (36.36%) | 226 (35.04%) | 88 (36.97%) | <.0001 |
| High | 14144 (41.93%) | 2313 (32.67%) | 1860 (29.64%) | 1407 (30.73%) | 559 (26.95%) | 285 (20.64%) | 174 (20.74%) | 51 (16.56%) | 30562 (49.75%) | 4876 (41.24%) | 3321 (38.53%) | 2375 (39.51%) | 757 (35.24%) | 418 (33.25%) | 226 (35.04%) | 81 (34.03%) | <.0001 |
| **EPICES deprivation score** |  |  |  |  |  |  |  |  |  |  |  |  |  |  |  |  | <.0001 |
| Low | 7815 (29.32%) | 2041 (38.17%) | 1826 (38.99%) | 1527 (44.83%) | 558 (36.73%) | 310 (31.66%) | 170 (27.24%) | 51 (21.25%) | 19617 (40.99%) | 4139 (46.68%) | 2799 (43.60%) | 1969 (43.63%) | 553 (34.31%) | 340 (37.57%) | 137 (27.85%) | 52 (30.41%) | (ref) |
| Intermediate | 7612 (28.56%) | 1344 (25.14%) | 1284 (27.42%) | 974 (28.60%) | 484 (31.86%) | 354 (36.16%) | 255 (40.87%) | 87 (36.25%) | 13993 (29.24%) | 2262 (25.51%) | 1613 (25.13%) | 1157 (25.64%) | 464 (28.78%) | 229 (25.30%) | 173 (35.16%) | 57 (33.33%) | 0.73 |
| High | 11229 (42.13%) | 1962 (36.69%) | 1573 (33.59%) | 905 (26.57%) | 477 (31.40%) | 315 (32.18%) | 199 (31.89%) | 102 (42.50%) | 14246 (29.77%) | 2466 (27.81%) | 2007 (31.27%) | 1387 (30.73%) | 595 (36.91%) | 336 (37.13%) | 182 (36.99%) | 62 (36.26%) | <.0001 |

SI conversion factors: To convert total cholesterol values to mmol/L, multiply by 0.0259; to convert glucose values to mmol/L, multiply by 0.0555

^a^ Individuals were matched by age and depression status

* Education and EPICES deprivation score were available from 2001 and 2003, respectively.

Note: All individuals with available cardiovascular health metrics were included in trend analyses for each specific metric; therefore, sample sizes might vary

by cardiovascular health metrics.

CVH = cardiovascular health

**e-Table 3.** Temporal trends in the prevalence of factors associated with cardiovascular health by sex and examination period

|  | **Women** | | | | **Men** | | | | **P-value**  **Interaction for sex^a^** |
| --- | --- | --- | --- | --- | --- | --- | --- | --- | --- |
| **Examination period** | **1992-1996** | **1997-2001** | **2002-2006** | **2007-2011** | **1992-1996** | **1997-2001** | **2002-2006** | **2007-2011** |  |
| Age (years) | 45.7 (13.6) | 45.7 (13.6) | 45.6 (13.6) | 45.6 (13.7) | 44.03 (12.1) | 44.03 (12.1) | 44.02 (12.1) | 44.02 (12.2) |  |
| Sex (%) | 38.04% (25917) | 38.04% (25917) | 38.04% (25917) | 38.04% (25917) | 61.96% (42221) | 61.96% (42221) | 61.96% (42221) | 61.96% (42221) |  |
| Smoking |  |  |  |  |  |  |  |  | <.0001 |
| Poor: Current | 24.55% (6322) | 26.42% (6847) | 24.50% (6349) | 20.43% (5293) | 30.27% (12693) | 35.05% (14798) | 33.65% (14209) | 32.62% (13771) | (ref) |
| Intermediate: Former or quit ≤12m | 2.10% (541) | 2.34% (606) | 1.72% (446) | 1.27% (330) | 3.50% (1468) | 3.53% (1492) | 2.24% (945) | 2.00% (846) | <.0001 |
| Ideal: Never or quit >12m ago | 73.35% (18888) | 71.24% (18463) | 73.78% (19121) | 78.30% (20288) | 66.23% (27773) | 61.41% (25927) | 64.11% (27067) | 65.37% (27595) | <.0001 |
| Body Mass Index |  |  |  |  |  |  |  |  | <.0001 |
| Poor: ≥30 kg/m^2^ | 7.34% (1903) | 9.09% (2354) | 13.19% (3391) | 18.39% (4668) | 7.33% (3093) | 9.52% (4019) | 10.50% (4411) | 11.46% (4766) | (ref) |
| Intermediate: 25-30 kg/m^2^ | 20.70% (5365) | 21.51% (5571) | 23.62% (6072) | 25.96% (6589) | 39.29% (16587) | 40.03% (16890) | 39.31% (16507) | 39.18% (16291) | <.0001 |
| Ideal: <25 kg/m^2^ | 71.96% (18648) | 69.40% (17972) | 63.19% (16247) | 55.65% (14123) | 53.39% (22541) | 50.45% (21288) | 50.19% (21075) | 49.36% (20523) | <.0001 |
| Physical Activity |  |  |  |  |  |  |  |  | 0.03 |
| Poor: No or walking <1 h/day | 67.61% (17519) | 56.10% (14539) | 58.08% (15052) | 61.11% (15827) | 59.95% (25308) | 51.24% (21636) | 53.94% (22773) | 54.25% (22892) | (ref) |
| Ideal: Walking >1 h/day | 32.39% (8392) | 43.90% (11378) | 41.92% (10865) | 38.89% (10074) | 40.05% (16904) | 48.76% (20585) | 46.06% (19448) | 45.75% (19308) | 0.03 |
| Total cholesterol |  |  |  |  |  |  |  |  | <.0001 |
| Poor: >6.138 mmol/L | 29.74% (7707) | 25.68% (6630) | 20.46% (5281) | 18.15% (4618) | 34.18% (14428) | 29.15% (12276) | 22.39% (9422) | 18.49% (7668) | (ref) |
| Intermediate: 5.136 – 6.138 mmol/L | 36.23% (9387) | 37.21% (9606) | 36.16% (9332) | 35.75% (9096) | 36.25% (15304) | 37.00% (15579) | 37.11% (15616) | 36.78% (15253) | <.0001 |
| Ideal: 5.136 mmol/L | 34.03% (8819) | 37.10% (9578) | 43.38% (11195) | 46.10% (11729) | 29.57% (12484) | 33.85% (14252) | 40.49% (17037) | 44.73% (18547) | <.0001 |
| Fasting glucose |  |  |  |  |  |  |  |  | <.0001 |
| Poor: >6.938 mmol/L | 2.17% (563) | 1.35% (350) | 1.72% (444) | 2.68% (684) | 4.87% (2054) | 2.82% (1189) | 2.85% (1200) | 3.50% (1453) | (ref) |
| Intermediate: 5.55- 6.938 mmol/L | 42.47% (11006) | 19.93% (5149) | 14.72% (3801) | 20.09% (5125) | 59.28% (25028) | 39.19% (16508) | 28.86% (12144) | 34.81% (14463) | <.0001 |
| Ideal: <5.55 mmol/L | 55.36% (14345) | 78.71% (20333) | 83.56% (21573) | 77.22% (19695) | 35.85% (15137) | 57.98% (24422) | 68.29% (28738) | 61.69% (25631) | <.0001 |
| Blood Pressure |  |  |  |  |  |  |  |  | <.0001 |
| Poor: ≥140/90 mmHg | 21.34% (5529) | 22.83% (5878) | 21.71% (5500) | 19.82% (4638) | 31.81% (13428) | 31.92% (13426) | 28.21% (11685) | 24.17% (9231) | (ref) |
| Intermediate: 120-139/80-89 mmHg | 57.31% (14851) | 39.79% (10246) | 35.02% (8871) | 35.79% (8375) | 62.03% (26185) | 48.73% (20493) | 46.46% (19246) | 50.82% (19412) | <.0001 |
| Ideal: <120/80 mmHg | 21.36% (5534) | 37.38% (9626) | 43.27% (10960) | 44.39% (10388) | 6.16% (2600) | 19.35% (8136) | 25.34% (10496) | 25.01% (9553) | <.0001 |
| Depressive symptoms: Yes | 11.40% (2955) | 11.40% (2955) | 11.40% (2955) | 11.40% (2955) | 5.36% (2263) | 5.36% (2263) | 5.36% (2263) | 5.36% (2263) |  |
| Education level* |  |  |  |  |  |  |  |  | 0.008 |
| Low |  | 13.39% (681) | 17.84% (4565) | 23.12% (5919) |  | 11.31% (968) | 14.54% (6083) | 17.92% (7486) | (ref) |
| Intermediate |  | 48.26% (2454) | 44.28% (11328) | 41.11% (10524) |  | 41.47% (3550) | 38.35% (16045) | 36.93% (15428) | 0.002 |
| High |  | 38.35% (1950) | 37.87% (9689) | 35.76% (9154) |  | 47.22% (4042) | 47.11% (19708) | 45.16% (18866) | 0.01 |
| EPICES deprivation score |  |  |  |  |  |  |  |  | 0.85 |
| Low |  |  | 37.26% (7036) | 29.56% (7262) |  |  | 46.56% (14340) | 38.13% (15266) | (ref) |
| Intermediate |  |  | 28.83% (5445) | 28.28% (6949) |  |  | 27.82% (8568) | 28.42% (11380) | 0.8 |
| High |  |  | 33.91% (6404) | 42.16% (10358) |  |  | 25.62% (7890) | 33.45% (13391) | 0.6 |

SI conversion factors: To convert total cholesterol values to mmol/L, multiply by 0.0259; to convert glucose values to mmol/L, multiply by 0.0555

^a^ Individuals were matched by age and depression status

* Education and EPICES deprivation score were available from 2001 and 2003, respectively.

Note: All individuals with available cardiovascular health metrics were included in trend analyses for each specific metric; therefore, sample sizes might vary

by cardiovascular health metrics.

CVH = Cardiovascular Health

**e-Table 4.** Percentage of women with and without children by age groups attending IPC visits

| **Age (years)** | **[20 - 29]** | **[30 - 39]** | **[40 - 49]** | **[50 - 59]** | **[60 - 69]** | **[70 - 79]** | **[80 - 89]** |
| --- | --- | --- | --- | --- | --- | --- | --- |
| **Women without children (%)** | 82.1 | 39.9 | 18.9 | 15.0 | 16.9 | 19.8 | 23.1 |
| **Women with children (%)** | 17.9 | 60.1 | 81.1 | 85.0 | 83.1 | 80.2 | 76.9 |

**e-Table 5.** HRs of women for All-Cause Mortality: analysis by age group^a^

|  | **N** | **event** | HRs (CIs) **^†^** | **p-value** |
| --- | --- | --- | --- | --- |
| **Total Population** |  |  |  |  |
| model 1 | 261863 | 14874 | 0.95 [0.92 - 0.99] | 0.0097 |
| model 2 | 105545 | 2419 | 0.82 [0.75 - 0.90] | <.0001 |
| **18-24 years** |  |  |  |  |
| model 1 | 12003 | 119 | 0.53 [0.35 - 0.81] | 0.004 |
| model 2 | 5041 | 19 | 0.34 [0.11 – 1.03] | 0.06 |
| **25-70 years** |  |  |  |  |
| model 1 | 242637 | 11953 | 0.83 [0.79 - 0.86] | <.0001 |
| model 2 | 97720 | 1950 | 0.71 [0.64 - 0.79] | <.0001 |

Reference group (men)

^a^ Individuals were matched on age and depression status
† The HRs and 95% CIs of each metric were estimated in separate Cox proportional hazards regression models.

model 1: Adjusted for cardiovascular risk factors

model 2: Adjusted for cardiovascular risk factors, depression status, and EPICES deprivation score

**e-Figure 1.** Flowchart of sample selection

**
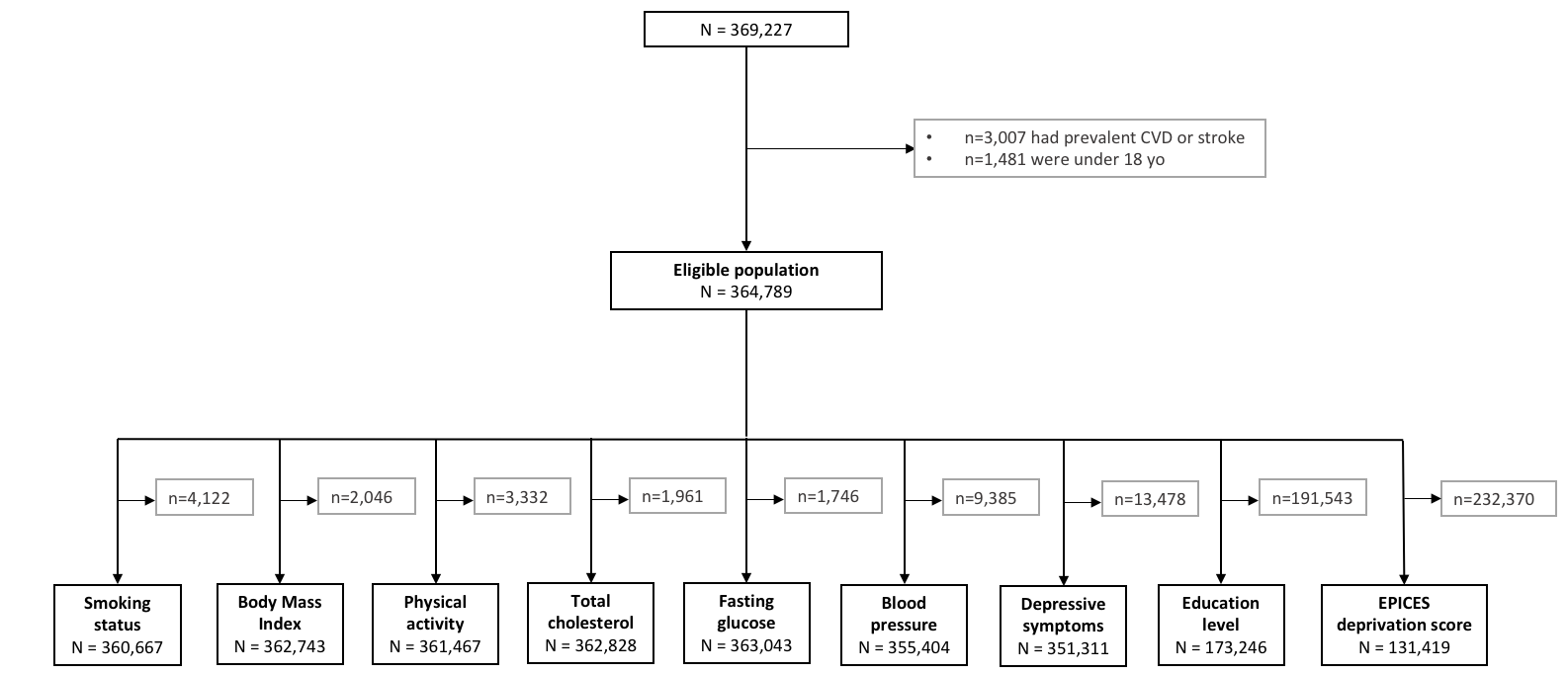
**

Horizontal arrows represent patients that were excluded from the eligible population.

**e-Figure 2.** Flowchart of sample selection matched by age and depressive status

**
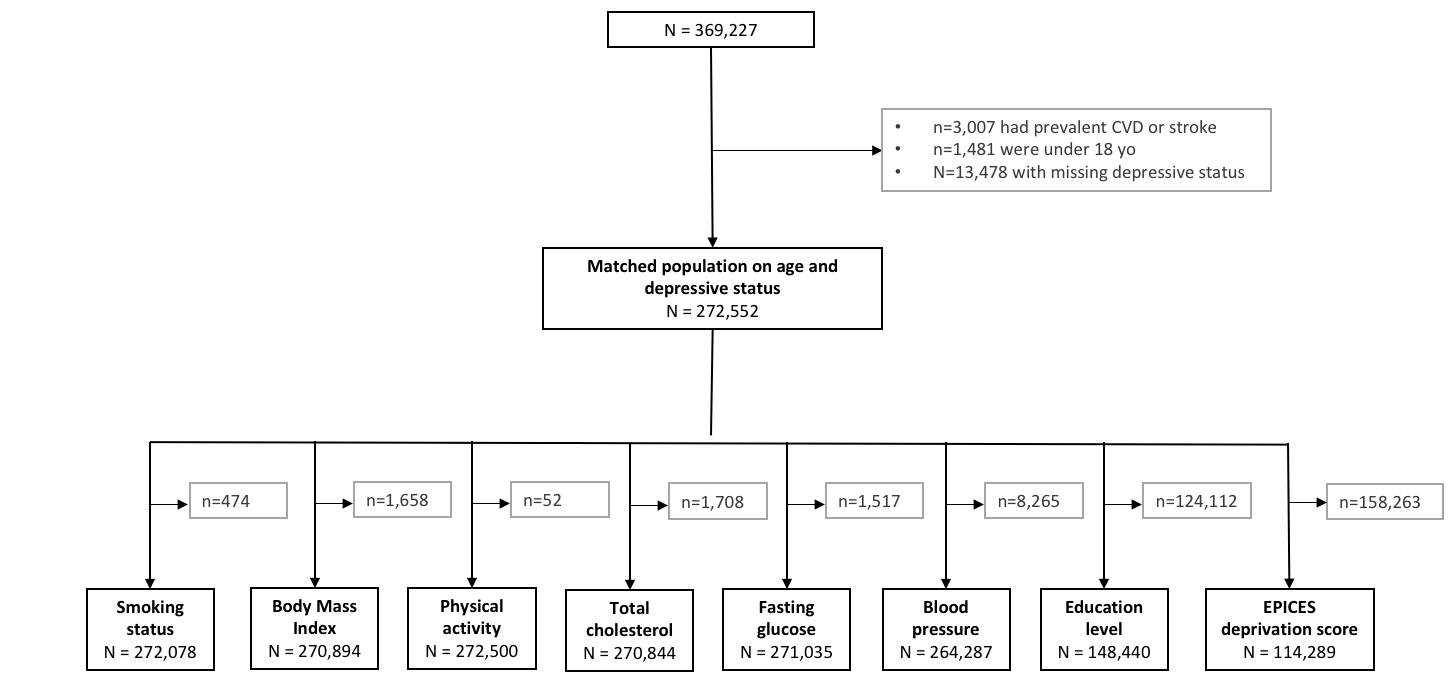
**

**e-Figure 3.** Kaplan Meier curves estimating the evolution of all-cause mortality over the study period by sex^a^

^a^ Individuals were matched on age and depression status
